# Cross-linked telopeptides of type I and III collagens in malignant ovarian tumours in vivo

**DOI:** 10.1038/sj.bjc.6690743

**Published:** 1999-10

**Authors:** S Kauppila, M K Bode, F Stenbäck, L Risteli, J Risteli

**Affiliations:** Departments of Clinical Chemistry, Pathology, University of Oulu, Oulu, FIN-90401, Finland

**Keywords:** collagen, ovarian neoplasms, invasion

## Abstract

Malignant tumours often induce a fibroproliferative response in the adjacent stroma, characterized by increased expression of type I and type III procollagens. In normal tissues, fibrillar collagens normally undergo extensive intermolecular cross-linking that provides tensile strength to the tissue. Here we set out to characterize collagen cross-linking in human ovarian carcinoma tissue in vivo. Biochemical and immunochemical methods were used for cross-linked telopeptides of type I and III collagens in samples of benign and malignant serous tumours. The locations and staining patterns of these proteins were visualized immunohistochemically. The contents of both total collagen and the cross-linked type I and type III collagens in the malignant samples were only about 20% of those in the benign tumours. The cross-linked telopeptide antigens derived from the collagens were smaller and more heterogeneous in size in the malignant than in the benign tumours, indicating a defective cross-linking process scarcely leading to the formation of mature cross-links in the collagen fibres in malignancy. Immunostaining revealed disorganized type I and type III collagen bundles in carcinomas. These findings suggest that the collagen cross-linking process is aberrant in malignant tumours, possibly resulting in increased susceptibility of tumour collagens for the proteolysis often associated with tumour invasion. © 1999 Cancer Research Campaign


*British Journal of Cancer* (1999) **81**(4), 654–661
© 1999 Cancer Research Campaign
Article no. bjoc.1999.0743

### Cross-linked telopeptides of type I and III collagens in malignant ovarian tumours in vivo

**S Kauppila[1], MK Bode[1], F Stenbäck[2], L Risteli[1] and J Risteli[1]**

Departments of [1]Clinical Chemistry and [2]Pathology, University of Oulu, FIN-90401 Oulu, Finland

**Summary** Malignant tumours often induce a fibroproliferative response in the adjacent stroma, characterized by increased expression of type I and type III procollagens. In normal tissues, fibrillar collagens normally undergo extensive intermolecular cross-linking that provides tensile strength to the tissue. Here we set out to characterize collagen cross-linking in human ovarian carcinoma tissue in vivo. Biochemical and immunochemical methods were used for cross-linked telopeptides of type I and III collagens in samples of benign and malignant serous tumours. The locations and staining patterns of these proteins were visualized immunohistochemically. The contents of both total collagen and the cross-linked type I and type III collagens in the malignant samples were only about 20% of those in the benign tumours. The cross-linked telopeptide antigens derived from the collagens were smaller and more heterogeneous in size in the malignant than in the benign tumours, indicating a defective cross-linking process scarcely leading to the formation of mature cross-links in the collagen fibres in malignancy. Immunostaining revealed disorganized type I and type III collagen bundles in carcinomas. These findings suggest that the collagen cross-linking process is aberrant in malignant tumours, possibly resulting in increased susceptibility of tumour collagens for the proteolysis often associated with tumour invasion. © 1999 Cancer Research Campaign

**Keywords:** collagen; ovarian neoplasms; invasion

Interaction between tumour cells and stromal components is important for the growth and invasion of a malignant tumour (Hauptmann et al, 1995; Iozzo, 1995). Alterations in the stroma of epithelial tumours involve the synthesis and degradation of extracellular matrix components and cell adhesion and migration (Liotta et al, 1990; Hart et al, 1992; Gui et al, 1995). Malignant tumours induce increased synthesis of type I and III procollagens resembling that of poorly controlled wound healing (Dvorak, 1986; Whalen, 1990). We have earlier shown that progressive ovarian carcinoma induces a fibroproliferative response characterized by active expression of type I and III procollagens (Zhu et al, 1993*a*, 1993*b*, 1995; Kauppila et al, 1996).

The fibre-forming type I and III collagens are synthesized as precursors, procollagens, with propeptide domains at both ends which are enzymatically cleaved off en bloc after their secretion into the extracellular matrix (Birk et al, 1990). The collagen molecules are incorporated into collagen fibres. The tensile strength of these fibres is due to covalent cross-links between several molecules within a fibre. The intermolecular cross-links are located in the short, non-triple-helical terminal parts known as telopeptides. The first reaction in cross-link formation is enzymatic, catalysed by lysyl oxidase, and then several spontaneous reactions lead to the formation of divalent cross-links, which mature into trivalent cross-links. The collagen cross-links in soft tissues are fully matured (Eyre et al, 1984; Yamauchi et al, 1988; Reiser et al, 1992). Interestingly, recent findings have shown that lysyl oxidase is similar to the tumour suppressor protein know as *rrg* (Contente et al, 1990; Kenyon et al, 1991) and its synthesis is decreased in malignant breast tumour tissue (Peyrol et al, 1997).

*Received 11 June 1998*
*Revised 15 February 1999*
*Accepted 10 March 1999*

*Correspondence to:* J Risteli

In this study we have analysed the cross-linking processes of type I and III collagen by analysing the collagen domains involved in intermolecular cross-linking. Specific immunoassays were used to measure the amount of the collagenous antigens directly in neutral salt extracts and in the insoluble residua of benign and malignant ovarian tumour tissues. In addition, the locations and staining intensities of type I and III collagens were studied in these samples by immunohistochemical (IHC) methods. We have been able to show – for the first time – that the cross-link maturation is defective in the malignant tissue in vivo.

### MATERIALS AND METHODS

### Tissue samples

Ovarian tumour tissue samples were obtained during operation at the Department of Obstetrics and Gynaecology, Oulu University Hospital. The diagnoses were based on histopathological analyses carried out at the Department of Pathology. The samples were frozen and stored at –20°C. Altogether 18 samples of benign serous cystadenomas and malignant serous cystadenocarcinomas were used in this study. The study was approved by the local Ethical Committee.

### Immunoassays for type I and type III collagens

The characteristics of the quantitative immunoassays used in this study are presented in Table 1. The concentrations of the type I collagen antigens, ICTP and PINP, were measured separately in duplicate by equilibrium radioimmunoassays (Orion Diagnostica, FIN-90460 Oulunsalo, Finland) with methods described previously (Risteli et al, 1993; Melkko et al, 1996). The ICTP assay detects trivalently cross-linked type I collagen in enzyme-digested

**Table 1** The immunoassays used for type I and type III collagen antigens

| Assay | Antibody raised against | Immunochemical specificity | Reference |
|---|---|---|---|
| ICTP | Trivalently cross-linked carboxyterminal telopeptide of human type I collagen | Trivalently cross-linked carboxyterminal telopeptide of type I collagen, weak reaction with divalently cross-linked and non-cross-linked type I collagen | Risteli et al, 1993 |
| IIINTP | Aminoterminal telopeptide of human type III collagen | Trivalently and divalently cross-linked type III collagen in tissue digests | Bode et al, 1999 |
| PINP | Intact aminoterminal propeptide of human type I procollagen | Type I procollagen, type I collagen with retained aminoterminal propeptide (= type I pN-collagen), intact aminoterminal propeptide | Melkko et al, 1996 |
| PIIINP | Aminoterminal propeptide of human type III procollagen | Type III procollagen, type III collagen with retained aminoterminal propeptide (= type III pN-collagen), intact aminoterminal propeptide | Risteli et al, 1988 |

tumour tissue, whereas the PINP assay was used to detect the intact aminoterminal propeptide of type I procollagen and such type I collagen that had retained aminoterminal propeptide (so-called type I pN-collagen) in soluble tissue extracts. The intra- and interassay coefficients of variation were 3–9% for PINP and 3–8% for ICTP. PINP, representing the intact, trimeric aminoterminal propeptide domain of type I procollagen, with a $M_r$ of about 35 000, was measured in tumour extracts using dilutions 1:1 to 1:10, which gave a linear inhibition in the radioimmunoassay. The ICTP concentrations were measured in enzyme-digested tumour samples using dilutions varying from 1:10 to 1:10 000, the former dilution for the malignant and the latter for the benign samples. The PIIINP assay was used to detect the intact aminoterminal propeptide of type III procollagen and type III pN-collagen in soluble tumour tissue extracts. The concentration of PIIINP was measured using an equilibrium radioimmunoassay giving intra- and interassay coefficients of variation 4–5% (Orion Diagnostica) (Risteli et al, 1988). The samples were diluted as for the PINP assay. In addition, PIIINP analysis was applied to the enzyme-digested tumour tissues.

The aminoterminal telopeptide of type III collagen, IIINTP, was measured using a novel immunomethod (Bode et al, 1999) in enzyme-digested tumour tissues. This method recognizes type III collagen domains involved in intermolecular cross-linking of type III collagen. The antigen was purified from human leiomyoma using a combination of Sephacryl S-300 gel exclusion chromatography and $C_{18}$ reverse phase chromatography on HPLC. The purified antigen was sequenced, and its purity and molecular size determined by sodium dodecyl sulphate polyacrylamide gel electrophoresis (SDS-PAGE) and gel exclusion chromatography. Polyclonal antibodies were raised in rabbits and one antiserum was selected for this study (AS #68). The dilution of the antiserum used in radioimmunoassay was 1:200, giving a 20% binding. 100 μl aliquots of a diluted sample were incubated with 200 μl of diluted antiserum and 200 μl of $^{125}$I-IIINTP solution for 2 h at 37°C. Then, 0.5 ml of a solution of the solid-phase second antibody was added and the tubes were incubated at 4°C for 30 min. They were centrifuged at 2000 ***g*** at 4°C for 30 min and the radioactivity in the precipitate was counted.

### Tissue preparation

For biochemical evaluation, the tissue samples of 0.4–3.6 g were cut in small pieces and phosphate–buffered saline (PBS)–Tween was added to 1 ml 100 mg$^{-1}$ of wet tissue weight. The samples were homogenized with an Ultra-Turrax homogenizer, cooled and let stand on ice for 30 min. They were then centrifuged at 12 000 ***g*** for 20 min. The supernatants of the extracted tumour tissue were collected for PINP and PIIINP analysis.

### Test for effectiveness of trypsin digestion

Prior to the analysis of the clinical series, the conditions for enzyme digestion were tested using both 0.1 mg and 1 mg trypsin (TPCK) per 100 mg of wet tissue sample. The samples were heat-denatured (+ 70°C for 1 h) and homogenized, and digestions were performed at + 37°C for 6 h. Then the samples were re-denatured, homogenized and retreated overnight with the same amount of newly added trypsin. ICTP and IIINTP were measured in the supernatant at different time points. The enzyme treatment was found to release the antigens during the first 2 h, and the total antigenicity was not altered during the next 18 h and the successive second enzyme treatment. Both concentrations of trypsin gave similar total amounts of recovered ICTP and IIINTP antigens, and the lower enzyme concentration was selected for further work.

### Enzyme treatment of the clinical samples

The insoluble pellets were suspended in 0.2 M $NH_4HCO_3$ (1 ml 100 mg$^{-1}$ of original wet tissue weight), denatured at + 70°C for 1 h before enzyme treatment, and treated with 0.1 mg of trypsin per 100 mg of the original wet tissue weight. Digestion was performed at + 37°C for 4 h; then the samples were re-denatured, homogenized and retreated with trypsin for another 4 h. After that, the samples were centrifuged at 2000 ***g*** for 30 min. The supernatants were used for ICTP, IIINTP and PIIINP analyses. For the ratio of PIIINP and IIINTP, the 50% loss in PIIINP antigenicity by trypsin treatment and the molecular sizes of both analytes were taken into account (Bode et al, 1999). The total collagen content of the tissue samples was measured by hydroxyproline analysis after acid hydrolysis (Kivirikko et al, 1967).

### Gel exclusion chromatography

Three trypsin-digested samples of benign disease and five of malignant disease were analysed by gel filtration. Then, 2 ml samples of the supernatants were applied to a Sephacryl S-100 column equilibrated in 0.2 M $NH_4HCO_3$ at room temperature, and 20-min fractions were collected with a flow rate of 5.4 ml h$^{-1}$. The apparent concentrations of ICTP, IIINTP and PIIINP were measured with the immunoassays as described above using an aliquot of the undiluted fraction or a 1:10 dilution. The fractions

*© 1999 Cancer Research Campaign* 

**Table 2** Contents of cross-linked type I and III collagen antigens (ICTP and IIINTP), and hydroxyproline in trypsin-digested ovarian tumours

| | ICTP | IIINTP | Hydroxyproline |
|---|---|---|---|
| Cystadenomas | 0.57 (0.44–0.71) mg g$^{-1}$ | 0.58 (0.33–0.83) mg g$^{-1}$ | 9.42 (5.63–13.21) mg g$^{-1}$ |
| (*n* = 8) | 100% | 100% | 100% |
| Carcinomas | 0.07 (0.04–0.11) mg g$^{-1}$ | 0.10 (0.05–0.14) mg g$^{-1}$ | 1.86 (0.94–2.75) mg g$^{-1}$ |
| (*n* = 10) | 13% | 17% | 20% |

The values present the mean (95% confidence intervals) as mg per tumour mass. The percentages indicate the relative values compared with cystadenomas.

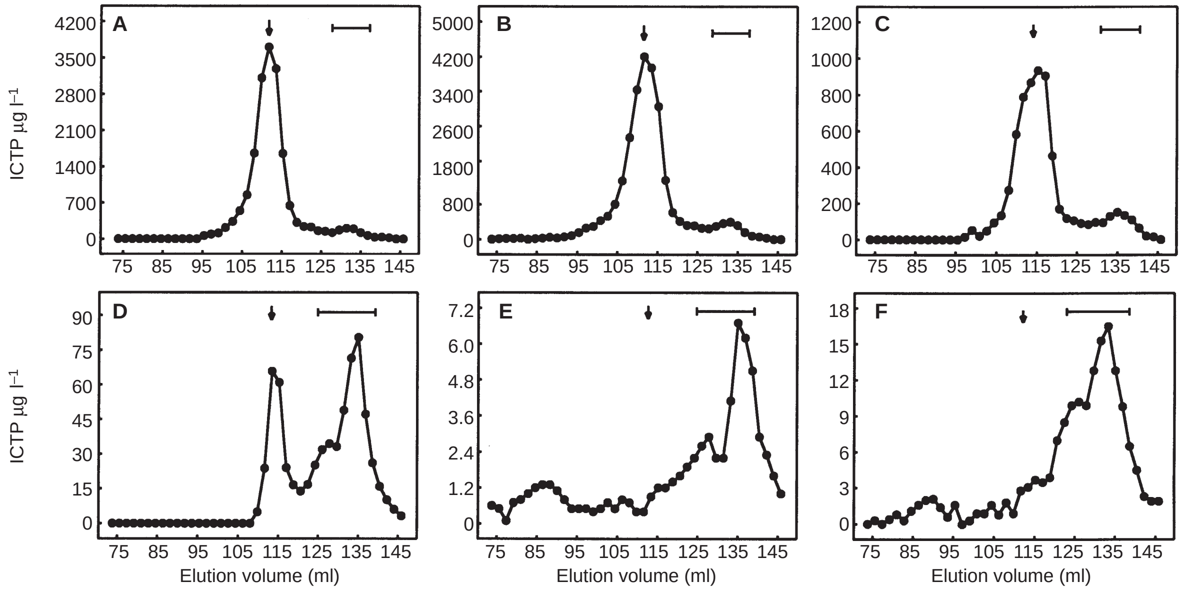

**Figure 1** Gel filtration analysis of the cross-linked carboxyterminal telopeptides of type I collagen in enzyme digested tumour tissue. (**A–C**) Benign ovarian cystadenoma; (**D–F**) malignant ovarian adenocarcinoma. The arrow indicates the elution position of the mature, trivalently cross-linked ICTP antigen and the bar those of immature, divalently cross-linked and non-cross-linked forms of ICTP. Note the difference in the ICTP scales between the benign and the malignant samples containing ICTP antigenicity and with the apparent molecular size of the trypsin-digested trivalent ICTP structure were pooled, freeze-dried and re-diluted in the volume of the original sample applied to the column. This separation was necessary to reliably estimate the content of mature, trivalent ICTP.

The supernatants of the extracted tumour tissues were also analysed on a Sephacryl S-300 column equilibrated in PBS–Tween. Twenty-minute fractions were collected with a flow rate of 6.0 ml h$^{-1}$ and the concentrations of PINP and PIIINP were analysed in the undiluted fractions with the immunoassays described.

### IHC analysis

Altogether 13 specimens from the same ovarian tumour samples as used above were obtained for IHC analysis. They included five samples of benign serous cystadenomas and eight samples of serous cystadenocarcinomas. The tissues were fixed in buffered neutral formalin, embedded in paraffin and sectioned at 5 µm. Immunohistochemical stainings were carried out using the avidin–biotin–immunoperoxidase technique after pepsin digestion (0.4% for 1–2 h at 37°C) as described previously in detail (Zhu et al, 1993*b*, 1995). The sections were stained with specific antibodies to the aminoterminal propeptides of type I and III procollagens, anti-PINP and anti-PIIINP respectively. An antibody to the cross-linked carboxyterminal telopeptide region of type I collagen, anti-ICTP, was used to visualize the cross-linked type I collagen fibres. These antibodies had been purified for IHC by cross-adsorption with several extracellular matrix proteins, and finally with the specific antigen in question coupled to CNBr-activated Sepharose 4B. Lack of cross-reaction with other antigens was tested by relevant radioimmuno-titration assays (Zhu et al, 1993*b*, 1995). The slides were counterstained with haematoxylin. The localizations and staining intensities of the different antigens in the tumour tissues were evaluated by a semi-quantitative scale based on visual estimation.

### Statistical analysis

Values are expressed as means (95% confidence intervals (CI)). Statistical analysis was carried out using the SPSS statistical analysis tool (for means and confidence intervals, and for Spearman's rank correlation analysis) and the CIA program for confidence interval analysis (for confidence intervals, and for Spearman's rank correlation).

 *© 1999 Cancer Research Campaign*

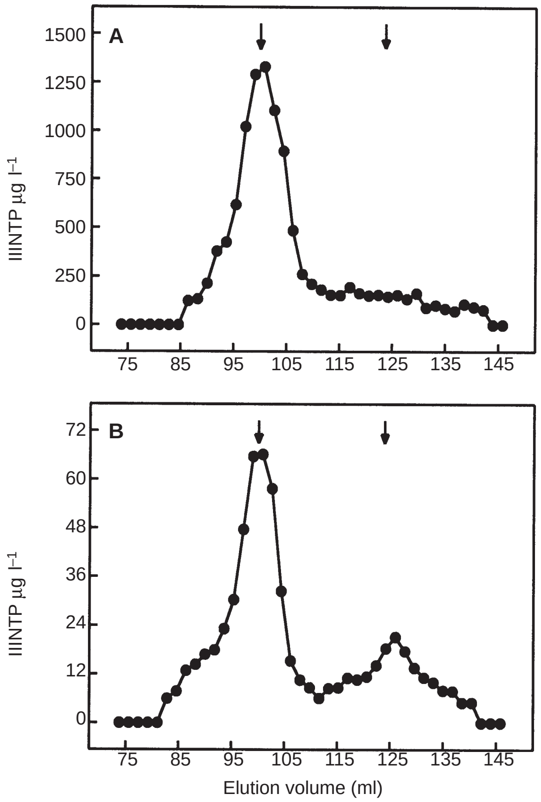

**Figure 2** Gel filtration analysis of the cross-linked aminoterminal telopeptide of type III collagen in enzyme digested tumour tissue. (**A**) Benign ovarian cystadenoma; (**B**) malignant ovarian adenocarcinoma. The first arrow indicates the elution position of the mature, trivalently cross-linked IIINTP and the second arrow that of immature, divalently cross-linked and non-cross-linked forms of IIINTP. Note the difference in the IIINTP scales between the benign and the malignant samples

**Table 3** Contents of type III pN-collagen in trypsin-digested ovarian tumours

| | PIIINP | PIIINP/IIINTP |
|---|---|---|
| Cystadenomas | 9.06 (6.11–12.01) µg $g^{-1}$ | 0.007 (0.004–0.010) |
| ($n$ = 8) | 100% | 100% |
| Carcinomas | 5.59 (3.91–7.27) µg $g^{-1}$ | 0.032 (0.020–0.044) |
| ($n$ = 10) | 62% | 454% |

The values present means (95% confidence intervals) as µg per tumour mass or as a ratio. The percentages indicate the relative values compared with cystadenomas. PIIINP = the aminoterminal propeptide of type III procollagen, IIINTP = the cross-linked aminoterminal telopeptide of type III collagen.

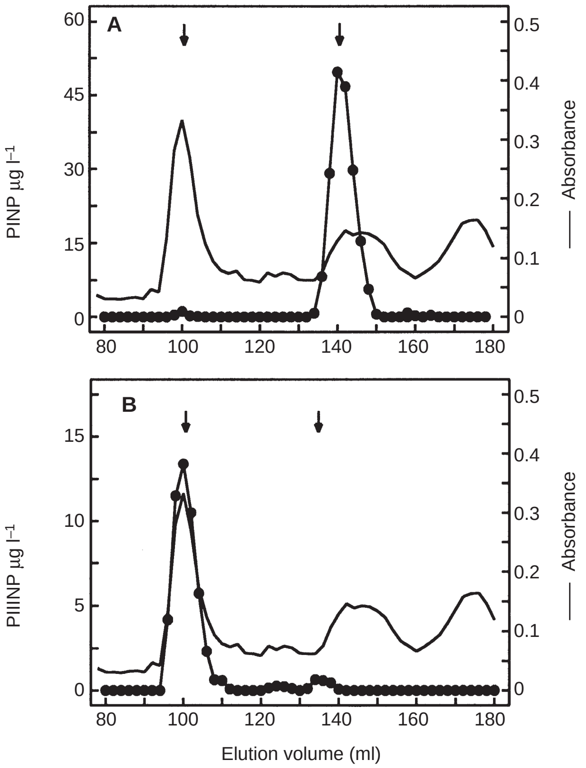

**Figure 3** Gel filtration analysis of type I and III procollagen synthesis. (**A**) PINP, (**B**) PIIINP in the soluble tissue extract of a malignant ovarian tumour. The first arrow indicates the elution positions of type I or type III pN-collagen, the second arrow that of the native aminoterminal propeptide of type I or type III procollagen

### RESULTS

### Insoluble collagens in ovarian tumours

The cross-linked, and thus insoluble, forms of type I and type III collagens were quantified by immunoassays that specifically detect the terminal telopeptide domains of the respective molecules containing a cross-link. Over 99% of the ICTP and IIINTP antigenicities were detected in the insoluble tissue fractions, and only 0.04–1% in the soluble form. The tissue contents of both ICTP and IIINTP were lower in the malignant than in the benign tissues. The benign cystadenomas showed an about 8 times higher ICTP content than did the adenocarcinomas (mean 0.57 mg $g^{-1}$ and 0.07 mg $g^{-1}$ respectively, Table 2).

4-Hydroxyproline, the hydroxylated amino acid responsible for the stability of the collagenous helix, makes up about 12% of the mass of a fibrillar collagen molecule and can be used as a general measure of the collagen content of a tissue. As expected, in the ovarian tumours most of the hydroxyproline was recovered in the insoluble fraction of the samples, with no difference between the tumour types in this respect. However, the collagen contents of the tissues varied greatly, with on the average 4.7 times more in the benign tumours (7.9% w/w) than in the adenocarcinomas (1.7% w/w) (Table 2).

The correlation between the hydroxyproline and ICTP contents was 0.98 ($P$ < 0.01, 95% CI 0.93–0.99) and that between the hydroxyproline and IIINTP contents 0.96 ($P$ < 0.01; 95% CI 0.88–0.98). There was also a strong correlation between ICTP and IIINTP ($r$ = 0.96; $P$ < 0.01; 95% CI 0.89–0.99).

The amounts of type I and type III collagens can be estimated from the contents of ICTP and IIINTP. In benign ovarian tissue the proportion of type III collagen was 35% (29–42%) of the sum of these collagens. Due to the defective cross-linking in type I collagen, in malignant tumours in particular (see below), such an estimate would not be accurate enough in carcinomas.

© 1999 Cancer Research Campaign

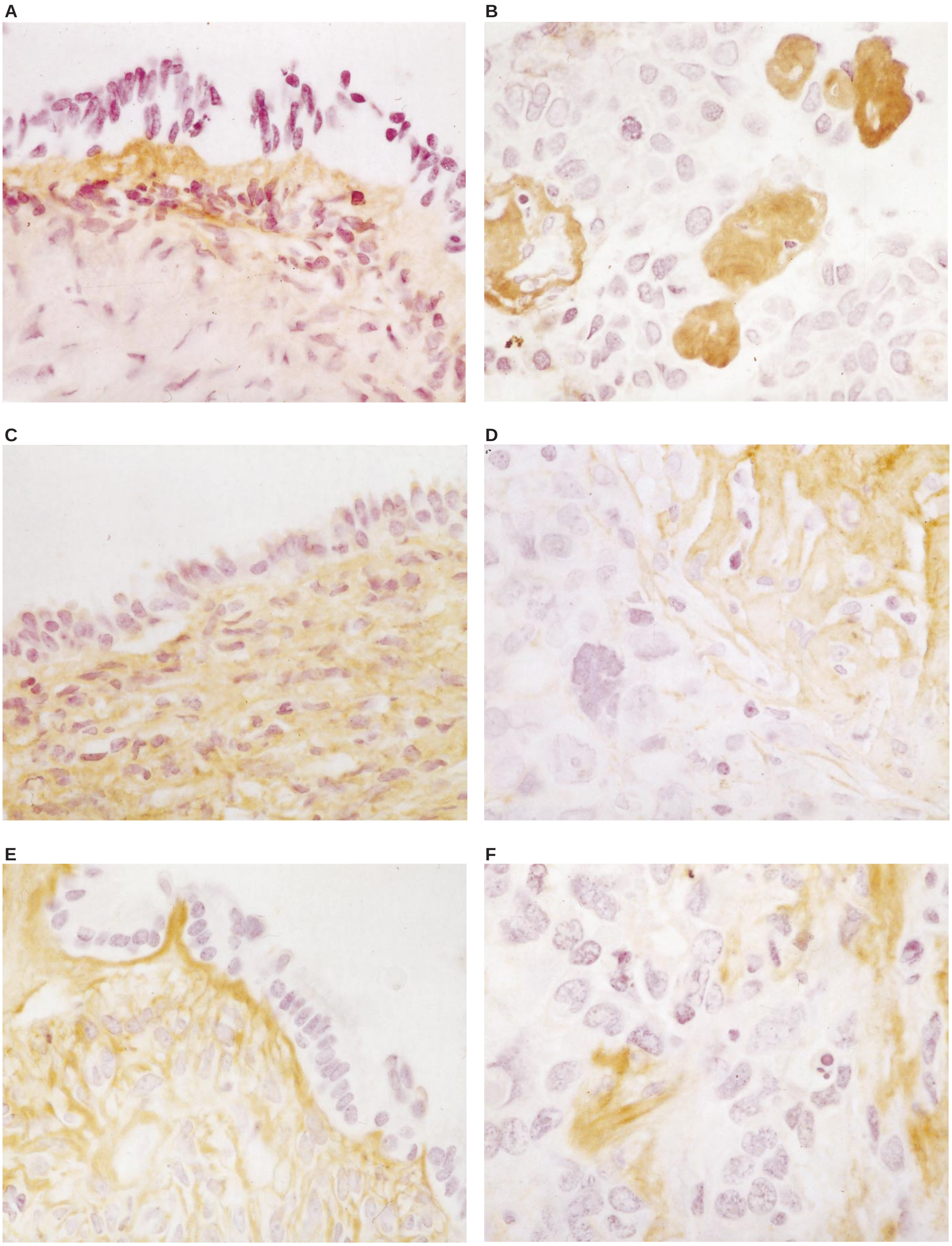

**Figure 4** Immunohistochemical staining of type I pN-collagen: (**A**) benign tumour; (**B**) malignant tumour, cross-linked type I collagen: (**C**) benign tumour; (**D**) malignant tumour, and type III pN-collagen; (**E**) benign tumour; (**F**) malignant tumour. Haematoxylin counterstain; original magnification, × 500

### Sizes of the cross-linked collagen antigens in digested tumour tissue

The sizes of the cross-linked telopeptide antigens were studied by gel filtration on Sepharose S-100 (Figures 1 and 2). In the benign tumours the ICTP antigen eluted in one major peak (Figure 1 A–C) corresponding in size to the trivalently cross-linked carboxyterminal telopeptide of type I collagen, as prepared by trypsin digestion. In contrast, the same antigenicity from the malignant tumours eluted mostly in two peaks with smaller molecular sizes, indicating a decreased proportion of the trivalently cross-linked ICTP

 *© 1999 Cancer Research Campaign*

domains (Figure 1 D–F). After removal of these immature, divalently cross-linked or non-cross-linked telopeptides, the decrease in the content of the trivalent ICTP in malignant samples is even more striking than in Table 2. The peak representing trivalent ICTP was pooled from the gel filtration runs of five malignant tumours, and the true ICTP content (mean 0.005 mg $g^{-1}$) in these pools was one-eighth of the apparent total ICTP content of the same tumours, analysed directly in the digests (mean 0.04 mg $g^{-1}$). These findings altogether indicate an about 70-fold difference in the content of trivalent ICTP between benign and malignant tumours, which is much greater than the difference in the hydroxyproline contents between the two groups.

A similar phenomenon, although less marked, was seen for the IIINTP antigen, which was heterogeneous both in the benign and the malignant samples, but in which the proportion of the less cross-linked species was larger in the carcinomas. Particularly in the malignant samples, three peaks – most probably representing trivalently and divalently cross-linked, and non-cross-linked forms of the IIINTP telopeptide – could be observed (Figure 2).

### Procollagen propeptides in ovarian tumour tissue

The aminoterminal propeptide domains of type I and III procollagens, the PINP and PIIINP antigens respectively, were quantified in the soluble fraction of the tissue samples. The mean PINP concentrations were higher in the carcinoma samples than in the benign tumours (2.45 (0.99–3.91) µg $g^{-1}$ and 1.00 (0.51–1.50) µg $g^{-1}$ respectively). Since only about half of the carcinoma samples had an increased PINP concentration, the difference between the means was not statistically significant. For PIIINP, no such tendency was evident between the benign and malignant tumours (2.04 (0.88–3.20) µg $g^{-1}$ and 1.44 (0.57–2.33) µg $g^{-1}$ respectively).

PIIINP was also assessed in the trypsin digests of the insoluble residues, whereas this was not possible for PINP because of the trypsin sensitivity of its antigenic epitope. In fact, about 80% of the PIIINP antigen was recovered in the insoluble fraction. There was no difference, however, between the total PIIINP contents in the benign and malignant tumours (Table 3). Since the PIIINP and IIINTP were assayed in the same samples, it is possible to estimate what part of type III collagen molecules retains the aminoterminal propeptide. In the benign tumours this was 0.7% (0.4–1.0) and in the malignant ones 3.2% (2.0–4.4) (Table 3).

### Sizes of the procollagen propeptide antigens in ovarian tumours

The soluble PINP and PIIINP antigens were characterized by gel filtration on Sepharose S-300. For both the benign and the malignant tumours, the major PINP peak eluted at a volume of 134–150 mL, which is the elution position of the intact, free trimeric aminoterminal propeptide of type I procollagen (Figure 3A). The majority of the PIIINP antigen, in contrast, eluted at 94–108 ml, corresponding to type III pN-collagen (Figure 3B). Again, there were only minor differences between the benign and the malignant samples.

### IHC studies of type I and III collagens

The following monospecific antibodies were used for visualizing the extracellular matrix structures in IHC: anti-PINP, to show type I pN-collagen; anti-ICTP, to show mature, cross-linked type I collagen; and anti-PIIINP, to show type III pN-collagen, which is present on the surface of practically all type III collagen fibres. In the benign tumours, all the type I and III collagen antigens had the appearance of regularly distributed collagen bundles (Figure 4 A, C and E). In the malignant tumours, both the staining intensities and the apparent distributions of the collagen bundles were more irregular (Figure 4 B, D and F).

Type I collagen staining, both for PINP and ICTP, was positive in all specimens. Type I pN-collagen staining was evident at the epithelial–stromal junction (Figure 4A) in all but one specimen. The more distant stroma stained only weakly in most of these specimens. Mature cross-linked type I collagen was seen both in the stroma adjacent to the epithelium and in the distant stroma of benign tumours (Figure 4C). In the poorly differentiated malignant tumours, aberrant PINP staining was seen in the vicinity of malignant cells (Figure 4B). The ICTP staining was slightly weaker in these areas (Figure 4D).

Type III pN-collagen was present at the epithelial–stromal junction in both the benign and the malignant tumours, often staining the collagen bundles in the normal appearing distant stroma (Figure 4E). In the stroma adjacent to the tumour cells its staining intensity varied from non-existent to strong. The difference between the staining intensities in the adjacent and distant stromas was less evident than for either of the type I collagen antigens.

### DISCUSSION

The total collagen content of the ovarian tissue was clearly lower in the malignant than in the corresponding benign tumours studied here. A similar difference was evident for the telopeptide domains of both major fibrillar collagen species. However, there was also a distinct qualitative difference between the telopeptide antigens of the benign and malignant tumour tissues, the latter containing remarkably less of the trivalently cross-linked ICTP and IIINTP antigens (Table 2).

Collagen fibres are responsible for the tensile strength of soft tissues. To fulfil this function, the individual rod-like molecules must be chemically cross-linked to their neighbours within the collagen fibril (Eyre et al, 1984; Yamauchi et al, 1988; Reiser et al, 1992). The cross-linking process involves a number of partially alternative reactions and in soft tissues normally leads to the formation of mature, trivalent cross-links within a relatively short time (Cronlund et al, 1985; Eyre et al, 1984). Only the first reaction in the collagen cross-linking pathway is catalysed by an enzyme, lysyl oxidase, whose expression by the fibroblasts generally parallels their collagen expression (Feres-Filho et al, 1996; Fushida-Takemura et al, 1996; Desmouliere et al, 1997). Despite the low collagen content in ovarian carcinoma tissue, the synthesis rates of both type I and III collagens are increased, as indicated in this study by the procollagen propeptide concentrations and by our previous in situ hybridization study (Kauppila et al, 1996). Thus there should be enough collagen substrate for the lysyl oxidase enzyme. Interestingly, it has recently been reported that lysyl oxidase expression is very low in breast carcinoma tissue (Peyrol et al, 1997) and that it is similar to the tumour suppressor protein *rrg* (Contente et al, 1990; Kenyon et al, 1991). Our finding of a defective collagen cross-linking process is in agreement with the assumption that the lack of this enzyme activity could be a general phenomenon in invading carcinomas. The disrupted collagen bundles visualized by our immunostainings could also be a result of defective cross-linking.

© 1999 Cancer Research Campaign

Since the telopeptide cross-links in benign ovarian tissues are fully matured, the total amounts of type I and III collagens can be reliably estimated from their contents of ICTP and IIINTP. Our results indicate that type III collagen makes up 35% of the sum of the type I and III collagens, which is about the same percentage, for example, as previously estimated for rat liver tissue by different methods (Kelley et al, 1984).

Collagen fibril formation takes place in the extracellular space immediately after the release of these proteins from the fibroblasts. The aminoterminal procollagen propeptides have been suggested to regulate the fibril formation (Fleischmajer et al, 1985). Our gel filtration findings confirm that the aminoterminal propeptide of type III procollagen frequently remains attached to the collagen molecule, whereas a significant part of the corresponding propeptide of type I procollagen is found in a free, cleaved form. In IHC this difference is supported by the fact that staining with anti-PIIINP antibodies can be usually detected throughout the stroma in benign tissue, whereas the PINP staining is mostly present in the junctional area next to the epithelium, where collagen synthesis is the most active. Interestingly, the relative amount of PIIINP when expressed over IIINTP in the insoluble matrix is 4.5-fold higher in the malignant than in the benign situation (Table 3), indicating an increased amount of newly synthesized type III collagen.

Although there needs not be a major difference in the tensile strength between the collagen fibres containing either divalent or trivalent cross-links (Eyre et al, 1984) such a difference can have other important effects. In irreversible fibroses of the liver and the skin (Ricard-Blum et al, 1993, 1996) the collagen contains more trivalent cross-links with the pyridinoline structure than does the collagen of the corresponding normal tissue. In this respect, ovarian carcinoma represents an opposite fibroproliferative situation with its decreased content of trivalently cross-linked collagens. It seems probable that defective cross-linking makes the collagens more susceptible for the degrading enzymes. This together with increased extracellular proteolysis in carcinomas, confirmed by several studies (Laiho et al, 1989; Van der Stappen et al, 1990; Aznavoorian et al, 1993; Stetler-Stevenson et al, 1993*a*, 1993*b*; Furcht et al, 1994; Stetler-Stevenson, 1996) could promote tumour invasion. The prognostic value of the defective collagen cross-linking in ovarian cancer remains to be elucidated, together with the possible presence of this phenomenon in other cancers.

### ACKNOWLEDGEMENTS

The authors gratefully acknowledge the expert technical assistance of Ms Päivi Annala, Ms Kristiina Apajalahti, Ms Riitta Karvonen, Ms Tuula Lujala, Mr Manu Tuovinen and Ms Mirja Vahera. This work was supported in part by research grants from the Finnish Cancer Foundations, the Technology Development Centre, Finland, and the Oulu University Hospital.

### REFERENCES

Aznavoorian S, Murphy AN, Stetler-Stevenson WG and Liotta LA (1993) Molecular aspects of tumor cell invasion and metastasis. *Cancer* **71**: 1368–1483

Birk DE, Zycband EI, Winkelmann DA and Trelstad RL (1990) Collagen fibrillogenesis in situ: discontinuous segmental assembly in extracellular compartments. *Ann NY Acad Sci* **580**: 176–194

Bode MK, Mosorin M, Satta J, Risteli L, Juvonen T and Risteli J (1999) Complete processing of type III collagen in atherosclerotic plaques. *Arterioscl Thromb Vasc Biol* **19**: 1506–1511

Contente S, Kenyon K, Rimoldi D and Friedman RM (1990) Expression of gene *rrg* is associated with reversion of NIH 3T3 transformed by LTR-c-H-*ras*. *Science* **249**: 796–798

Cronlund AL, Smith BD and Kagan HM (1985) Binding of lysyl oxidase to fibrils of type I collagen. *Connect Tissue Res* **14**: 109–119

Desmouliere A, Darby I, Costa AM, Raccurt M, Tuchweber B, Sommer P and Gabbiani G (1997) Extracellular matrix deposition, lysyl oxidase expression, and myofibroblastic differentation during the initial stages of cholestatic fibrosis in the rat. *Lab Invest* **76**: 765–778

Dvorak HF (1986) Tumors: wounds that do not heal: similarities between tumor stroma generation and wound healing. *N Engl J Med* **315**: 1650–1659

Eyre DR, Paz MA and Gallop PM (1984) Cross-linking in collagen and elastin. *Ann Rev Biochem* **53**: 717–748

Feres-Filho EJ, Menassa GB and Trackman PC (1996) Regulation of lysyl oxidase by basic fibroblast growth factor in osteoblastic MC3T3-E1 cells. *J Biol Chem* **271**: 6411–6416

Fleischmajer R, Perlish JS and Timpl R (1985) Collagen fibrillogenesis in human skin. *Ann NY Acad Sci* **460**: 246–257

Furcht LT, Skubitz APN and Fields GB (1994) Tumor cell invasion, matrix metalloproteinases, and the dogma. *Lab Invest* **70**: 781–783

Fushida-Takemura H, Fukuda M, Maekawa N, Chanoki M, Kobayashi H, Yashiro N, Ishii M, Hamada T, Otani S and Ooshima A (1996) Detection of lysyl oxidase gene expression in rat skin during wound healing. *Arch Dermatol Res* **288**: 7–10

Gui GPH, Puddefoot JR, Vinson GP, Wells CA and Carpenter R (1995) In vitro regulation of human breast cancer cell adhesion and invasion via integrin receptors to the extracellular matrix. *Br J Surg* **82**: 1192–1196

Hart IR and Saini A (1992) Biology of tumour metastasis. *Lancet* **339**: 1453–1457

Hauptmann S, Zardi L, Siri A, Carnemolla B, Borsi L, Castellucci M, Klosterhalfen B, Hartung P, Weis J, Stöcker G, Haubeck H-D and Kirkpatrick CJ (1995) Extracellular matrix proteins in colorectal carcinomas. Expression of tenascin and fibronectin isoforms. *Lab Invest* **73**: 172–182

Iozzo RV (1995) Tumor stroma as a regulator of neoplastic behavior. *Lab Invest* **73**: 157–160

Kauppila S, Saarela J, Stenbäck F, Risteli J, Kauppila A and Risteli L (1996) Expression of mRNAs for type I and type III procollagens in serous ovarian cystadenomas and cystadenocarcinomas. *Am J Pathol* **148**: 539–548

Kelley J, Chrin L and Evans JN (1984) Microquantitation of insoluble tissue collagen (Types I and III) by radiodilution assay. *Anal Biochem* **139**: 115–125

Kenyon K, Contente S, Trackman PC, Tang J, Kagan HM and Friedman RM (1991) Lysyl oxidase and *rrg* messenger RNA? *Science* **253**: 802

Kivirikko KI, Laitinen O and Prockop DJ (1967) Modifications of a specific assay for hydroxyproline in urine. *Anal Biochem* **19**: 249–255

Laiho M and Keski-Oja J (1989) Growth factors in the regulation of pericellular proteolysis: a review. *Cancer Res* **49**: 2533–2553

Liotta LA and Kohn E (1990) Cancer invasion and metastases. *JAMA* **263**: 1123–1126

Melkko J, Kauppila S, Niemi S, Risteli L, Haukipuro K, Jukkola A and Risteli J (1996) Immunoassay for intact aminoterminal propeptide of human type I procollagen. *Clin Chem* **42**: 947–954

Peyrol S, Raccurt M, Gerard F, Gleyzal C, Grimaud JA and Sommer P (1997) Lysyl oxidase gene expression in the stromal reactions to in situ and invasive ductal breast carcinoma. *Am J Pathol* **150**: 497–507

Reiser K, McCormic RJ and Rucker RB (1992) Enzymatic and nonenzymatic cross-linking of collagen and elastin. *FASEB J* **6**: 2439–2449

Ricard-Blum S, Bresson-Hadni S, Guerret S, Grenard P, Volle P-J, Risteli L, Grimaud J-A and Vuitton DA (1996) Mechanism of collagen network stabilization in human irreversible granulomatous liver fibrosis. *Gastroenterology* **111**: 172–182

Ricard-Blum S, Esterre P and Grimaud JA (1993) Collagen cross-linking by pyridinoline occurs in non-reversible skin fibrosis. *Cell Mol Biol* **39**: 723–727

Risteli J, Niemi S, Trivedi P, Mäentausta O, Mowat AP and Risteli L (1988) Rapid equilibrium radioimmunoassay for the aminoterminal propeptide of human type III procollagen. *Clin Chem* **34**: 715–718

Risteli J, Elomaa I, Niemi S, Novamo A and Risteli L (1993) Radioimmunoassay for the pyridinoline cross-linked carboxy-terminal telopeptide of type I collagen: a new serum marker of bone collagen degradation. *Clin Chem* **39**: 635–640

Stetler-Stevenson WG (1996) Dynamics of matrix turnover during pathologic remodeling of the extracellular matrix. *Am J Pathol* **148**: 1345–1350

Stetler-Stevenson WG, Aznavoorian S and Liotta LA (1993*a*) Tumor cell interactions with the extracellular matrix during invasion and metastasis. *Annu Rev Cell Biol* **9**: 541–573

 © 1999 Cancer Research Campaign

Stetler-Stevenson WG, Liotta LA and Kleiner DE (1993*b*) Extracellular matrix 6: role of matrix metalloproteinases in tumor invasion and metastasis. *FASEB J* **7**: 1434–1441
Van der Stappen JWJ, Hendriks T and Wobbes T (1990) Correlation between collagenolytic activity and grade of histological differentiation in colorectal tumors. *Int J Cancer* **45**: 1071–1078
Whalen GF (1990) Solid tumours and wounds: transformed cells misunderstood as injured tissue? *Lancet* **336**: 1489–1492
Yamauchi M and Mechanic GL (1988) Cross-linking of collagen. In: *Collagen*, Nimni ME (ed), pp. 157–172. CRC Press: Boca Raton
Zhu G-G, Risteli J, Puistola U, Kauppila A and Risteli L (1993*a*) Progressive ovarian carcinoma induces synthesis of type I and type III procollagens in the tumor tissue and peritoneal cavity. *Cancer Res* **53**: 5028–5032
Zhu G-G, Stenäck F, Risteli L, Risteli J, Kauppila A (1993*b*) Organization of type III collagen in benign and malignant ovarian tumors. *Cancer* **72**: 1679–1684
Zhu G-G, Risteli L, Mäkinen M, Risteli J, Kauppila A, Stenbäck F (1995) Immunohistochemical study of type I collagen and type I pN-collagen in benign and malignant ovarian neoplasms. *Cancer* **75**: 1010–1017

*© 1999 Cancer Research Campaign*

## References

[PDF_00606] Aznavoorian S., Murphy A. N., Stetler-Stevenson W. G., Liotta L. A. (1993). Molecular aspects of tumor cell invasion and metastasis.. Cancer.

[PDF_00608] Birk D. E., Zycband E. I., Winkelmann D. A., Trelstad R. L. (1990). Collagen fibrillogenesis in situ. Discontinuous segmental assembly in extracellular compartments.. Ann N Y Acad Sci.

[PDF_00611] Bode M. K., Mosorin M., Satta J., Risteli L., Juvonen T., Risteli J. (1999). Complete processing of type III collagen in atherosclerotic plaques.. Arterioscler Thromb Vasc Biol.

[PDF_00614] Contente S., Kenyon K., Rimoldi D., Friedman R. M. (1990). Expression of gene rrg is associated with reversion of NIH 3T3 transformed by LTR-c-H-ras.. Science.

[PDF_00617] Cronlund A. L., Smith B. D., Kagan H. M. (1985). Binding of lysyl oxidase to fibrils of type I collagen.. Connect Tissue Res.

[PDF_00619] Desmoulière A., Darby I., Costa A. M., Raccurt M., Tuchweber B., Sommer P., Gabbiani G. (1997). Extracellular matrix deposition, lysyl oxidase expression, and myofibroblastic differentiation during the initial stages of cholestatic fibrosis in the rat.. Lab Invest.

[PDF_00623] Dvorak H. F. (1986). Tumors: wounds that do not heal. Similarities between tumor stroma generation and wound healing.. N Engl J Med.

[PDF_00625] Eyre D. R., Paz M. A., Gallop P. M. (1984). Cross-linking in collagen and elastin.. Annu Rev Biochem.

[PDF_00627] Feres-Filho E. J., Menassa G. B., Trackman P. C. (1996). Regulation of lysyl oxidase by basic fibroblast growth factor in osteoblastic MC3T3-E1 cells.. J Biol Chem.

[PDF_00630] Fleischmajer R., Perlish J. S., Timpl R. (1985). Collagen fibrillogenesis in human skin.. Ann N Y Acad Sci.

[PDF_00632] Furcht L. T., Skubitz A. P., Fields G. B. (1994). Tumor cell invasion, matrix metalloproteinases, and the dogma.. Lab Invest.

[PDF_00634] Fushida-Takemura H., Fukuda M., Maekawa N., Chanoki M., Kobayashi H., Yashiro N., Ishii M., Hamada T., Otani S., Ooshima A. (1996). Detection of lysyl oxidase gene expression in rat skin during wound healing.. Arch Dermatol Res.

[PDF_00638] Gui G. P., Puddefoot J. R., Vinson G. P., Wells C. A., Carpenter R. (1995). In vitro regulation of human breast cancer cell adhesion and invasion via integrin receptors to the extracellular matrix.. Br J Surg.

[PDF_00641] Hart I. R., Saini A. (1992). Biology of tumour metastasis.. Lancet.

[PDF_00642] Hauptmann S., Zardi L., Siri A., Carnemolla B., Borsi L., Castellucci M., Klosterhalfen B., Hartung P., Weis J., Stöcker G. (1995). Extracellular matrix proteins in colorectal carcinomas. Expression of tenascin and fibronectin isoforms.. Lab Invest.

[PDF_00646] Iozzo R. V. (1995). Tumor stroma as a regulator of neoplastic behavior. Agonistic and antagonistic elements embedded in the same connective tissue.. Lab Invest.

[PDF_00648] Kauppila S., Saarela J., Stenbäck F., Risteli J., Kauppila A., Risteli L. (1996). Expression of mRNAs for type I and type III procollagens in serous ovarian cystadenomas and cystadenocarcinomas.. Am J Pathol.

[PDF_00651] Kelley J., Chrin L., Evans J. N. (1984). Microquantitation of insoluble tissue collagen (types I and III) by radiodilution assay.. Anal Biochem.

[PDF_00653] Kenyon K., Contente S., Trackman P. C., Tang J., Kagan H. M., Friedman R. M. (1991). Lysyl oxidase and rrg messenger RNA.. Science.

[PDF_00655] Kivirikko K. I., Laitinen O., Prockop D. J. (1967). Modifications of a specific assay for hydroxyproline in urine.. Anal Biochem.

[PDF_00657] Laiho M., Keski-Oja J. (1989). Growth factors in the regulation of pericellular proteolysis: a review.. Cancer Res.

[PDF_00659] Liotta L. A., Kohn E. (1990). Cancer invasion and metastases.. JAMA.

[PDF_00661] Melkko J., Kauppila S., Niemi S., Risteli L., Haukipuro K., Jukkola A., Risteli J. (1996). Immunoassay for intact amino-terminal propeptide of human type I procollagen.. Clin Chem.

[PDF_00664] Peyrol S., Raccurt M., Gerard F., Gleyzal C., Grimaud J. A., Sommer P. (1997). Lysyl oxidase gene expression in the stromal reaction to in situ and invasive ductal breast carcinoma.. Am J Pathol.

[PDF_00667] Reiser K., McCormick R. J., Rucker R. B. (1992). Enzymatic and nonenzymatic cross-linking of collagen and elastin.. FASEB J.

[PDF_00669] Ricard-Blum S., Bresson-Hadni S., Guerret S., Grenard P., Volle P. J., Risteli L., Grimaud J. A., Vuitton D. A. (1996). Mechanism of collagen network stabilization in human irreversible granulomatous liver fibrosis.. Gastroenterology.

[PDF_00673] Ricard-Blum S., Esterre P., Grimaud J. A. (1993). Collagen cross-linking by pyridinoline occurs in non-reversible skin fibrosis.. Cell Mol Biol (Noisy-le-grand).

[PDF_00679] Risteli J., Elomaa I., Niemi S., Novamo A., Risteli L. (1993). Radioimmunoassay for the pyridinoline cross-linked carboxy-terminal telopeptide of type I collagen: a new serum marker of bone collagen degradation.. Clin Chem.

[PDF_00676] Risteli J., Niemi S., Trivedi P., Mäentausta O., Mowat A. P., Risteli L. (1988). Rapid equilibrium radioimmunoassay for the amino-terminal propeptide of human type III procollagen.. Clin Chem.

[PDF_00685] Stetler-Stevenson W. G., Aznavoorian S., Liotta L. A. (1993). Tumor cell interactions with the extracellular matrix during invasion and metastasis.. Annu Rev Cell Biol.

[PDF_00683] Stetler-Stevenson W. G. (1996). Dynamics of matrix turnover during pathologic remodeling of the extracellular matrix.. Am J Pathol.

[PDF_00692] Stetler-Stevenson W. G., Liotta L. A., Kleiner D. E. (1993). Extracellular matrix 6: role of matrix metalloproteinases in tumor invasion and metastasis.. FASEB J.

[PDF_00698] Whalen G. F. (1990). Solid tumours and wounds: transformed cells misunderstood as injured tissue?. Lancet.

[PDF_00702] Zhu G. G., Risteli J., Puistola U., Kauppila A., Risteli L. (1993). Progressive ovarian carcinoma induces synthesis of type I and type III procollagens in the tumor tissue and peritoneal cavity.. Cancer Res.

[PDF_00708] Zhu G. G., Risteli L., Mäkinen M., Risteli J., Kauppila A., Stenbäck F. (1995). Immunohistochemical study of type I collagen and type I pN-collagen in benign and malignant ovarian neoplasms.. Cancer.

[PDF_00705] Zhu G. G., Stenbäck F., Risteli L., Risteli J., Kauppila A. (1993). Organization of type III collagen in benign and malignant ovarian tumors. An immunohistochemical study.. Cancer.

[PDF_00695] van der Stappen J. W., Hendriks T., Wobbes T. (1990). Correlation between collagenolytic activity and grade of histological differentiation in colorectal tumors.. Int J Cancer.

